# American ginseng with different processing methods ameliorate immunosuppression induced by cyclophosphamide in mice via the MAPK signaling pathways

**DOI:** 10.3389/fimmu.2023.1085456

**Published:** 2023-04-21

**Authors:** Yan-Ting Zhang, Wei Tian, Yu-Shun Lu, Zhi-Man Li, Duo-Duo Ren, Yue Zhang, Ji-Yue Sha, Xiao-Hui Huo, Shan-Shan Li, Yin-Shi Sun

**Affiliations:** ^1^ Institute of Special Animal and Plant Sciences, China Academy of Agricultural Sciences, Changchun, China; ^2^ Institute of Cash Crops, Hebei Academy of Agricultural and Forestry Sciences, Shijiazhuang, China; ^3^ Institute of Biological and Pharmaceutical Engineering, Jilin Agricultural Science and Technology University, Jilin, China

**Keywords:** American ginseng, steam-processing, CTX, immunosuppression, MAPK

## Abstract

This study aimed to clarify the effects of two processed forms of American ginseng (*Panax quinquefolius* L.) on immunosuppression caused by cyclophosphamide (CTX) in mice. In the CTX-induced immunosuppressive model, mice were given either steamed American ginseng (American ginseng red, AGR) or raw American ginseng (American ginseng soft branch, AGS) by intragastric administration. Serum and spleen tissues were collected, and the pathological changes in mice spleens were observed by conventional HE staining. The expression levels of cytokines were detected by ELISA, and the apoptosis of splenic cells was determined by western blotting. The results showed that AGR and AGS could relieve CTX-induced immunosuppression through the enhanced immune organ index, improved cell-mediated immune response, increased serum levels of cytokines (TNF-α, IFN-γ, and IL-2) and immunoglobulins (IgG, IgA, and IgM), as well as macrophage activities including carbon clearance and phagocytic index. AGR and AGS downregulated the expression of BAX and elevated the expression of Bcl-2, p-P38, p-JNK, and p-ERK in the spleens of CTX-injected animals. Compared to AGS, AGR significantly improved the number of CD4^+^CD8^-^T lymphocytes, the spleen index, and serum levels of IgA, IgG, TNF-α, and IFN-γ. The expression of the ERK/MAPK pathway was markedly increased. These findings support the hypothesis that AGR and AGS are effective immunomodulatory agents capable of preventing immune system hypofunction. Future research may investigate the exact mechanism to rule out any unforeseen effects of AGR and AGS.

## 1 Introduction

The immune system, consisting of immune organs, immune cells, and cytokine (CK), is a critical defense of the body. To protect the body from numerous external antigens, allergens, and infections while preserving normal physiological homeostasis, specific and non-specific immunities are triggered by self and foreign substances under normal physiological conditions (1). However, this balance can be affected by genetic factors, environmental factors, age, gender, physical stress, mental stress, dietary habits, etc., leading to immunological imbalance and disease (2). Since 1958, solid carcinomas, hematological malignancies, autoimmune disorders, and other diseases have all been treated with cyclophosphamide (CTX), an alkylating drug with immunosuppressive properties (3, 4). CTX is inactive as a precursor drug *in vitro* and functions primarily *in vivo* via hepatic P450 enzyme hydrolysis to aldophosphamide, which subsequently enters the tissues to create phosphoramide mustard (5). By interfering with DNA and RNA functions, cross-linking DNA, and obstructing DNA synthesis, CTX affects the cell cycle and, thus, inhibits the proliferation of T and B lymphocytes (6). The active intermediates of CTX cannot distinguish between normal and malignant cells in this process. CTX often causes myelosuppression and immunosuppression, as evidenced by leukopenia, neutropenia, decreased lymphocyte proliferation, and decreased cytokine production (7). As a result, CTX is frequently utilized to establish immunosuppressed mice models for studying the effects of immunosuppression on various diseases and testing the efficacy of new immunosuppressive drugs.

The primary method of therapy in traditional medical systems is herbal, which is now extensively employed in clinics. Important species of *Panax* L. used to cure various ailments include notoginseng, Asian ginseng, and American ginseng (8, 9). In RAW 264.7 mouse macrophages, Azike et al. found that American ginseng extracts greatly increased the expression of TNF-α and IL-6 while showing an immunostimulatory effect (10, 11). The first line of defense against microbial infections is innate immunity, which is mediated by macrophages and kills pathogens through phagocytosis or the production of cytokines such as TNF-α. As reported by Yu et al., American ginseng extracts significantly improved the phagocytosis of mice abdominal macrophages (12). To treat intestinal immunological diseases in mice, Zhou et al. found that American ginseng extracts could heal damaged intestinal mucosa by increasing the variety and amount of beneficial intestinal flora (13). American ginseng can be used to treat and prevent colitis by increasing the expression of iNOS and COX-2 while decreasing the expression of p53 (14). Additionally, American ginseng reduced the expression of COX-2 and NF-κB (15), increased the expression of EGFR, decreased proliferation, and increased apoptosis (16). Thus, it was confirmed that American ginseng has various pharmacological effects such as immunomodulation, anti-aging (17), anti-inflammatory, and cancer prevention.

It has been extensively reported that ginseng undergoes significant changes in chemical composition and biological activity after steam-processing (18–21), and such changes in biological effects may be related to the steam-processing treatment. In the past, raw sun ginseng dominated the market for American ginseng products. In recent years, a large number of steamed American ginsengs have entered the market at a much higher price than raw sun ginseng. In addition, it has been reported that the content of polar ginsenosides in American ginseng significantly decreased after steaming, while the content of less polar ginsenosides increased accordingly, generating new valuable compounds (22, 23). The antiproliferative effect of ginseng on HT-29 human colorectal cancer cells was significantly increased after steaming and could be increased by extending the steaming time within a certain range (24). Heat processing may disrupt the cell wall of American ginseng and release the antioxidant compounds within it, which inhibits lipid peroxidation and increases the activity of antioxidant enzymes, resulting in more significant antioxidant activity of steamed American ginseng (25). However, no comparison has been made between the immunological activities of American ginseng before and after processing. In this study, we proposed using BALB/c mice to evaluate the differences in immunomodulatory activities between steamed American ginseng (AGR) and unsteamed American ginseng (AGS), investigate the effect of steaming treatment, and reveal its mechanism of action. The findings could provide data support for a comprehensive evaluation of the nutritional quality of American ginseng and provide a basis for its consumption to prevent and treat coronavirus disease 2019 (COVID-19).

## 2 Materials and methods

### 2.1 Materials and reagents

Mouse tumor necrosis factor α (TNF-α) kit (YJ002095), mouse interleukin 2 (IL-2) kit (YJ02295), mouse interferon γ (IFN-γ) kit (YJ002277), mouse immunoglobulin G (IgG) kit (ml037601), mouse immunoglobulin A (IgA) kit (ml037606), and mouse immunoglobulin M (IgM) kit (ml063597) were purchased from Shanghai Enzyme Link Biotechnology Co., Ltd. (Shanghai, China). The 10% neutral formalin (SL1560) was purchased from Beijing Coolaber Technology Co., Ltd. (Beijing, China). P38α/β (sc-7972), ERK (sc-7383), JNK (sc-7345), p-JNK (sc-6254), p-P38 (sc-7973), and p-ERK (sc-7383) were purchased from Santa Cruz Biotechnology, Inc. (CA, USA). Bcl-2 (ab182858), BAX (ab81083), and GAPDH (ab8245) were obtained from Abcam (Cambridge, MA, USA). RPMI-1640 medium (C11875500BT), fetal bovine serum (FBS, 164210), and Thermo Life Penicillin Streptomycin Sol (15070063) were obtained from Thermo Fisher Scientific Co., Ltd. (Shanghai, China); CTX (H32020857) was purchased from Shengdi Pharmaceutical Co., Ltd. (Jiangsu, China). Levamisole Hydrochloride Tablets (H37020819) were purchased from Renhe Tang Pharmaceutical Co. Ltd. (Linyi, China). PE/Cyanine7 anti-mouse CD3 (100220), FITC anti-mouse CD4 (100509), and APC anti-mouse CD8a (100712) were obtained from BioLegend (San Diego, CA, USA). ConA (C8110) was purchased from Beijing Solarbio Science and Technology Co., Ltd. (Beijing, China).

### 2.2 American ginseng sources and preparation

Fresh samples were collected from Weihai, Shandong Province, China, and identified as *Panax quinquefolius* L. (4 years old) by Professor Wei Li of the School of Chinese Herbal Medicine, Jilin Agricultural University. AGR was prepared as follows. Briefly, 500 g of fresh samples were steamed at 98°C for 2 h. After steaming, the ginseng was dried in a desiccator at 60°C for 24 h. The process was repeated 8 times (26, 27). AGS was prepared with 500 g of fresh samples. After washing and drying, the samples were dried at 25°C for 48 h. Then, the temperature was raised 2°C/2 day to 35°C and raised to 40°C after the main ginseng body was softened. After drying for 48 h, the temperature was lowered to 34°C until completely dry. The pulverized AGR and AGS were mixed separately with water and refluxed for 2 h. The process was repeated 3 times, and the residues were removed. The 3 extracts were combined, concentrated, freeze-dried, and then stored in a cool, dry environment.

### 2.3 CTX-induced immunosuppression

#### 2.3.1 Determination of body weight and organ indices

**Figure 1:**
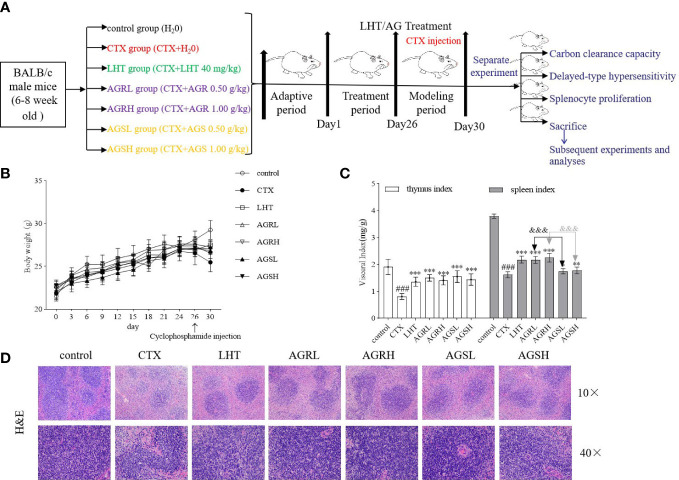
Effects of AGR and AGS on body weight and immune organ indices in mice. Except for the control group, mice were administered CTX through intraperitoneal injection for 4 consecutive days since day 26. Mice were euthanized by cervical dislocation on day 31, blood was collected, and the spleen and thymus were isolated. The spleen and thymus were weighed, and organ indices were calculated. **(A)** shows the overall therapeutic procedural design scheme. **(B)**, body weight (N = 8, means ± SEM); **(C)** immune organ index (N = 8, means ± SEM); **(D)**, spleen histopathology in mice (scale bar = 200 μm, objective: 10×; scale bar = 50 μm, objective: 40×). All data shown are representative of three independent experiments with similar results. The statistical significance was analyzed using one-way ANOVA. CTX, cyclophosphamide-induced immunosuppressive group; LHT, levamisole hydrochloride group; AGRL, American ginseng red low dose group; AGRH, American ginseng red high dose group; AGSL, American ginseng soft branch low dose group; AGSH, American ginseng soft branch high dose group. ^###^ *P* < 0.001 vs. Control group, ^*^ *P* < 0.05 vs. CTX group, ^**^ *P* < 0.01 vs. CTX group, ^***^ *P* < 0.001 vs. CTX group, ^&&&^ *P* < 0.001 vs. AGS group.

Liaoning Changsheng Biotechnology Co. Ltd. provided male BALB/c mice (SPF level, License No. (Liao)-2020-0001). The guiding principle was followed during the care and use of mice. The experiment was approved by the Experimental Animal Ethics Committee of the Institute of Specialties, Chinese Academy of Agricultural Sciences (Changchun, China) (Permit No. ISAPSAEC-2022-78). Seven groups were prepared after a 7-day acclimation period: control group, CTX group, LHT group (levamisole hydrochloride, 40 mg/kg), AGR low dose group (0.50 g/kg, AGRL), AGR high dose group (1.00 g/kg, AGRH), AGS low dose group (0.50 g/kg, AGSL), and AGS high dose group (1.00 g/kg, AGSH). In this experiment, the intragastric administration method was used, which means that the drug solution or suspension is instilled with a device (gavage needle) directly into the end of the esophagus or stomach of the mice. In contrast, oral administration involves mixing the drug with food or dissolving it in drinking water and allowing the animal to ingest it freely (28). Although this method is simpler and more convenient to use, it does not guarantee the accuracy of the drug dose and therefore does not objectively reflect the experimental results. The intragastric route is often used to mimic a common dosing route in humans. It also allows for precise dosing of substances when compared to oral administration through food or water (29). The mice from the AGR groups and AGS groups were given different doses of AGR and AGS every day, while the control group mice and the CTX group mice was intragastrically administered an equal amount of normal saline. All mice other than the control group were administered CTX (50 mg/kg) through intraperitoneal injection for 4 consecutive days since day 26. After the last administration, some mice were used for the determination of carbon clearance, delayed hypersensitivity reaction and spleen cell proliferation rate, respectively (**Figure 1A**). The remaining mice were euthanized, and blood and spleen were collected for subsequent experiments and analysis.

#### 2.3.2 Histological examination

The spleen tissues were fixed with 10% paraformaldehyde, rinsed with water for 1 h, dehydrated and transparent with ethanol, embedded in wax by immersion, trimmed in wax blocks, and serially sectioned at 4 μm thickness. After dewaxing, sections were stained with hematoxylin and eosin, and histopathological changes in the spleen were observed under an Olympus BH22 microscope.

### 2.4 Cellular immunity experiments

#### 2.4.1 ConA-induced splenocyte proliferation

Spleens were collected from the sacrificed mice under aseptic conditions and then gently crushed and lysed of red blood cells to create a splenocyte solution on day 31. The cells were washed 3 times with PBS, and the cell concentration was adjusted to 3×10^6^ cells/mL with RPMI-1640 complete medium. The splenocyte suspension was separated into two wells of a 24-well culture plate, one with 75 µL of ConA solution and the other as a control, before incubating at 37°C and 5% CO_2_ for 68 h. At the end of incubation, MTT (5 mg/mL) was added to each well, and the incubation continued for 4 h at 37°C and 5% CO_2_. To dissolve the purple precipitate, 1 mL of acidic isopropanol solution was added to each well at the end of the incubation. The wells were then dispersed into 96-well culture plates, and the optical density (OD) was determined. The optical density values of the wells with ConA were subtracted from the optical density values of the wells without ConA to represent the proliferation capacity of lymphocytes.

#### 2.4.2 Sheep red blood cells induced delayed-type hypersensitivity

On day 30, 0.2 mL of 2% defibrinated SRBC (1×10^8^ cells) was injected intraperitoneally 1 h after CTX administration to stimulate the proliferation of T lymphocytes into sensitized lymphocytes in mice. Based on our earlier work (30), footpad thickness was measured using vernier calipers on day 31, and the footpad was attacked with SRBC and measured again after 24 h. The changes in footpad thickness before and after the reaction correspond to the degree of DTH and indicate the effect of American ginseng on cellular immunity.

### 2.5 Carbon clearance capacity

Each mouse received an intravenous injection of 4 times diluted India ink at a dose of 0.1 mL/10 g) on day 31. At 2 min (t_1_) and 10 min (t_2_) afterward, 20 μl blood was collected from the retinal venous plexuses and immediately mixed with 2 ml of 0.1% Na_2_CO_3_. The absorbance was measured at 600 nm in an ELISA reader. Mice were euthanized by cervical dislocation, and their spleen and liver were removed and weighed. Phagocytic index a was used to express the carbon clearance capacity of the mice, which can be calculated according to the following equation.


$$K=\frac{\log OD1-\log OD2}{t2-t1}$$

$$a=\frac{\text{Body weight}}{Liver\;weight+Spleen\;weight}\times \sqrt[{3}]{K}$$

### 2.6 Measurement of splenic t-lymphocyte subpopulations and leukocyte counts

The ocular venous plexuses of mice were sampled for 20 µL of blood, and leukocytes were quantified using a hemocytometer on day 31. Then, the animals were euthanized, and sterile spleens were gently crushed and lysed of erythrocytes to make spleen cell suspension. Based on the experimental procedure in our previous study (31), the cell concentration was adjusted to 1×10^6^ cells/mL, and splenocyte surface markers were labeled with fluorescein isothiocyanate (FITC)-coupled anti-mouse CD4, APC-coupled anti-mouse CD8a, and PerCP-Cy5-coupled anti-mouse CD3. The cells were maintained at room temperature for 40 min under natural light, washed twice, resuspended with 5 mL PBS, and analyzed using the FACSCalibur and CellQuest software.

### 2.7 Cytokines detection by ELISA

Blood samples were collected from the ophthalmic vein plexus of mice and centrifuged at 4°C for 10 min. The serum was stored at -80°C on day 31. The contents of various cytokines and immunoglobulins were determined according to the instructions of the ELISA kit.

### 2.8 Western blotting

Protein was extracted from spleen homogenate using lysis buffer. Protein concentration was determined using the BCA method. In addition, 10% to 15% SDS-PAGE gels were prepared to separate equal amounts of proteins. Proteins were transferred to PVDF membranes under the constant voltage of 70 V. After membrane transfer, 5% skim milk powder was added and incubated for 1 h. Subsequently, membranes were washed by PBST for 30 min. Then, specific primary antibodies, including Bax, Bcl-2, p ERK, p-JNK, p-P38, ERK, P38, JNK, and GAPDH, were added before incubating overnight at 4°C. The primary antibody was washed off by PBST, and an HRP-coupled secondary antibody was added for incubation on a slow-shaking shaker at room temperature for 1 h. After washing off the secondary antibody, the target proteins were visualized using the BeyoECL Plus kit, and Western Blot bands were determined using Image G image analysis. The net gray value was determined by Image G image analysis and compared with the internal reference GAPDH assay, and the ratio was calculated to compare the differences between the groups.

### 2.9 Statistical analysis

Results are presented as mean ± SEM. Statistical tests were performed using GraphPad Prism 9.0. One-way analysis of variance (One-way ANOVA test) was used to test the differences between groups, and *P<* 0.05 indicated significant differences.

## 3 Results

### 3.1 Effect of AGR and AGS on body weight and immune organ indices

During the test period, the mice in each test group had increased body weights, with no significant difference from the control group (**Figure 1B**). Mice in the CTX group had significantly lower body weight, thymic indices (**Figures 1B, C**), and spleen indices compared to mice in the control group. LHT, AGR, and ARS groups had considerably increased body weight, thymic indices, and spleen indices compared to the CTX group. Compared to the AGS group, spleen indices were considerably higher in the AGR group, indicating that the steamed American ginseng increased the immune organ index of immunocompromised mice. H&E staining showed that the control spleen’s red pulp (RP) and white pulp (WP) were clear with obvious boundaries, and the splenic corpuscle (SCor) was apparent (**Figure 1D**). The RP and WP were not clearly defined, and the lymph sheath around the tiny central artery was weakened in CTX-induced immunosuppressive mice, indicating that CTX may have harmed the splenic immune cells. The RP and WP were demarcated after AGR and AGS treatments, and the marginal area of the WP was expanded, demonstrating that AGR and AGS could heal CTX-induced spleen damage.

### 3.2 Effect of AGR and AGS on monocyte-macrophage function and cellular immunity

**Figure 2:**
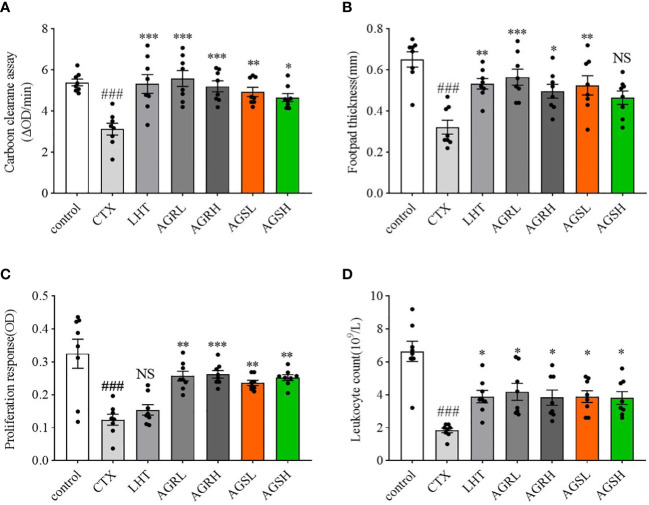
Effects of AGR and AGS on ConA-induced splenocyte proliferation, SRBC-induced DTH, carbon clearance capacity, and leukocytes in CTX-induced immunosuppressive mice. Except for the control group, the mice were administered CTX through intraperitoneal injection for 4 consecutive days since day 26. **(A)**, carbon clearance capacity (N = 8, means ± SEM). Each mouse received an intravenous injection of 4 times diluted India ink on day 31. At 2 min and 10 min afterward, 20 μl blood was collected from the retinal venous plexuses and mixed with 2 ml of 0.1% Na_2_CO_3_ at once. The absorbance was measured at 600 nm in an ELISA reader. Mice were euthanized by cervical dislocation, and their spleens and livers were removed and weighed; **(B)**, SRBC-induced DTH (N = 8, means ± SEM). On day 30, 1 h after CTX administration, a certain dose of defibrinated SRBC was injected intraperitoneally into mice to stimulate the proliferation of T lymphocytes into sensitized lymphocytes. Footpad thickness was measured using vernier calipers on day 31, and the footpad was attacked with SRBC and measured again 24 h later; **(C)**, ConA-induced splenocyte proliferation (N = 8, means ± SEM). The animals were euthanized and sterile spleens were gently crushed and lysed of erythrocytes to make spleen cell suspension. Induction of splenocyte proliferation was achieved using a certain dose of ConA; **(D)**, leukocyte count (N = 8, means ± SEM). Blood was collected from the ocular venous plexus of the mice, and leukocytes were quantified using a hemocytometer on day 31. All data shown are representative of three independent experiments with similar results. The statistical significance was analyzed using one-way ANOVA. CTX, cyclophosphamide-induced immunosuppressive group; LHT, levamisole hydrochloride group; AGRL, American ginseng red low dose group; AGRH, American ginseng red high dose group; AGSL, American ginseng soft branch low dose group; AGSH, American ginseng soft branch high dose group. ^###^ *P* < 0.001 vs. Control group, ^*^ *P* < 0.05 vs. CTX group, ^**^ *P* < 0.01 vs. CTX group, ^***^ *P* < 0.001 vs. CTX group, NS: no significant difference.

By reflecting the phagocytic activity of the macrophages with the carbon clearance capacity, the function of non-specific immunity can be assessed. As illustrated in **Figure 2A**, the carbon particle clearance rate was significantly lower in the CTX group compared to the control group. The carbon particle clearance rate was significantly higher in the LHT, AGR, and AGS groups compared to the CTX group. DTH is a T lymphocyte-mediated hypersensitive reaction that can be used to detect the immunological performance of cellular immunity. The footpad thickness in the CTX group was lower than the control group (**Figure 2B**), while those of the LHT, AGR, and AGS groups tended to rise. Compared with the CTX group, the footpad thicknesses in the AGRL, AGRH, and AGSL groups were significantly increased. ConA is a mitogen for T lymphocytes, and it selectively stimulates lymphocyte proliferation (32). To measure the level of cellular immunity, we used ConA-induced splenocyte proliferation. Compared to the control group, splenocyte proliferation was significantly decreased in the CTX group. Compared to the CTX group, splenocyte proliferation was significantly higher in the AGR and AGS groups (**Figure 2C**). Compared to the control group, leukocyte counts were significantly lower in the CTX group; leukocyte counts were significantly higher in the LHT, AGR, and AGS groups than in the CTX group (**Figure 2D**). AGR and AGS may improve CTX-induced immunosuppression in mice by increasing the number of leukocytes, macrophage phagocytosis, and T-lymphocyte proliferation capacity.

### 3.3 Effect of AGR and AGS on splenic T-lymphocyte subpopulations

**Figure 3:**
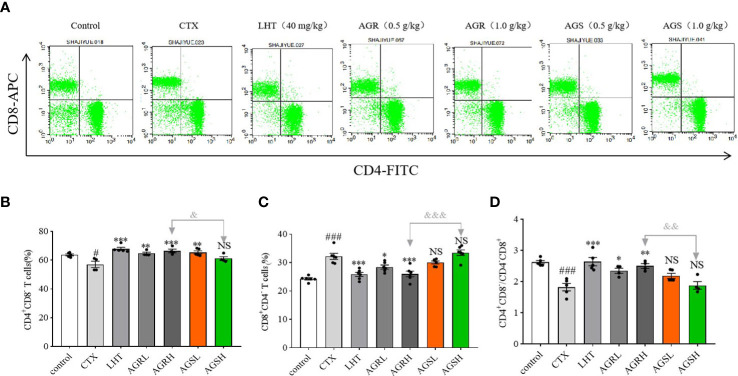
Effects of AGR and AGS on the proportion of splenic CD4 or CD8T cells. Except for the control group, the mice were administered CTX through intraperitoneal injection for 4 consecutive days since day 26. Then, the animals were euthanized and sterile spleens were gently crushed and lysed of erythrocytes to make spleen cell suspension. Splenocyte surface markers were labeled with fluorescein isothiocyanate (FITC)-coupled anti-mouse CD4, APC-coupled anti-mouse CD8a, and PerCP-Cy5-coupled anti-mouse CD3. The cells were maintained at room temperature for some time under natural light and analyzed using FACSCalibur and CellQuest software. **(A)**, flow cytometry; **(B)**, the proportion of splenic CD4^+^CD8^-^ T cells (N = 4-5, means ± SEM); **(C)**, the proportion of splenic CD4^-^CD8^+^ T cells (N = 8, means ± SEM); **(D)**, the ratio of splenic CD4/CD8 T cells (N = 8, means ± SEM). All data shown are representative of three independent experiments with similar results. The statistical significance was analyzed using one-way ANOVA. CTX, cyclophosphamide-induced immunosuppressive group; LHT, levamisole hydrochloride group; AGRL, American ginseng red low dose group; AGRH, American ginseng red high dose group; AGSL, American ginseng soft branch low dose group; AGSH, American ginseng soft branch high dose group. ^#^ *P* < 0.05 vs. Control group, ^###^ *P* < 0.001 vs. Control group, ^*^ *P* < 0.05 vs. CTX group, ^**^ *P* < 0.01 vs. CTX group, ^***^ *P* < 0.001 vs. CTX group, ^&^ *P* < 0.05 vs. AGR group, ^&&^ *P* < 0.01 vs. AGS group, ^&&&^ *P* < 0.001 vs. AGS group, NS: no significant difference.

As T cell proliferation is required for specific immune activation, we conducted a phenotypic analysis of the total T cells and the T cell subsets (**Figure 3**). The content of CD4^+^CD8^-^T cells and the ratio of CD4^+^CD8^-^/CD4^-^CD8^+^ were significantly downregulated in the CTX group compared to the control group. Compared with the CTX group, the content of CD4^+^CD8^-^T cells and the ratio of CD4^+^CD8^-^/CD4^-^CD8^+^ in the LHT, AGRL, AGRH, and AGSL groups were significantly upregulated. Compared to the AGS group, the spleen lymphocyte content of CD4^+^CD8^-^T cells and the CD4^+^CD8^-^/CD4^-^CD8^+^ ratio were significantly higher in the AGR group, while the content of CD4^-^CD8^+^T cells was significantly lower. The balance of T-lymphocyte subpopulations in mice was restored by steamed American ginseng.

### 3.4 Effect of AGR and AGS on serum levels of immunoglobulins and cytokines

**Figure 4:**
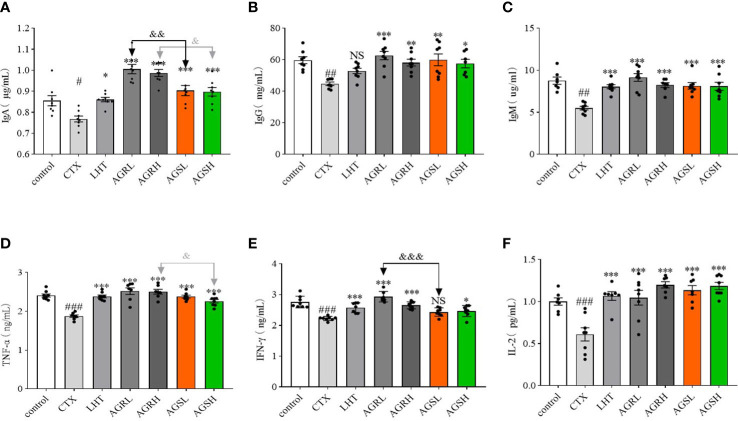
The immunoglobulins and cytokines in CTX-induced immunosuppressive mice. Except for the control group, the mice were administered CTX through intraperitoneal injection for 4 consecutive days since day 26. Blood samples were collected from the ophthalmic vein plexus of mice on day 31 and centrifuged at 4°C for 10 min, and the serum was stored at -80°C. The contents of various cytokines and immunoglobulins were determined according to the instructions of the ELISA kit (N = 8, means ± SEM). **(A)**, IgA; **(B)**, IgG; **(C)**, IgM; **(D)**, TNF-α; **(E)**, IFN-γ; **(F)**, IL-2. All data shown are representative of three independent experiments with similar results. The statistical significance was analyzed using one-way ANOVA. CTX, cyclophosphamide-induced immunosuppressive group; LHT, levamisole hydrochloride group; AGRL, American ginseng red low dose group; AGRH, American ginseng red high dose group; AGSL, American ginseng soft branch low dose group; AGSH, American ginseng soft branch high dose group. ^#^ *P* < 0.05 vs. Control group, ^##^ *P* < 0.01 vs. Control group, ^###^ *P* < 0.001 vs. Control group, ^*^ *P* < 0.05 vs. CTX group, ^**^ *P* < 0.01 vs. CTX group, ^***^ *P* < 0.001 vs. CTX group, ^&^ *P* < 0.05 vs. AGS group, ^&&^ *P* < 0.01 vs. AGS group, ^&&&^ *P* < 0.001 vs. AGS group, NS: no significant difference.

We used ELISA kits to detect the serum levels of immunoglobulins (IgA, IgG, and IgM) and cytokines (TNF-α, IFN-γ, and IL-2) and examine the effects of American ginseng aqueous extracts on CTX-induced immunosuppression. Compared to the control group, the expression of IgA, IgG, IgM, TNF-α, IFN-γ, and IL-2 fractions were decreased in the CTX group (**Figures 4A–F**). Compared with the CTX group, the expression of IgA, IgM, TNF-α, IFN-γ, and IL-2 was significantly increased in the LHT group, the expression of IgA, IgG, IgM, TNF-α, IFN-γ, and IL-2 was significantly increased in the AGR group, and the expression of IgA, IgG, IgM, TNF-α, IFN-γ, and IL-2 was significantly increased in the AGS group. The steamed American ginseng improved the immune system of immunocompromised mice by increasing the expression of immunoglobulins and cytokines.

### 3.5 Effect of AGR and AGS on apoptosis

**Figure 5:**
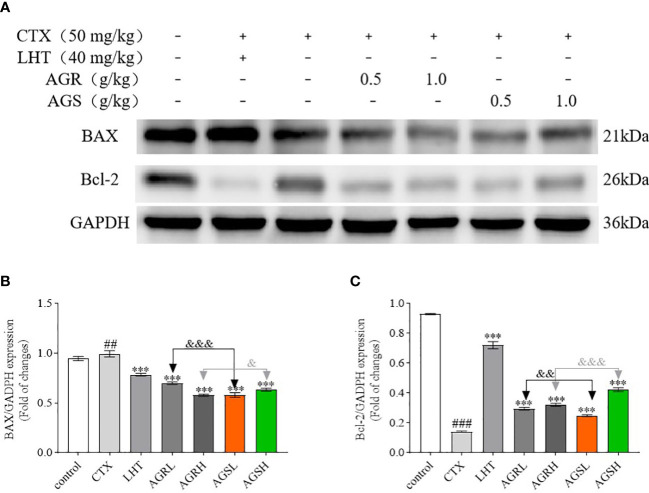
The protein expression of BAX and Bcl-2 in the spleen (N = 8, means ± SEM). **(A)**, images of the bands from the Western blotting; **(B)**, the expression of BAX/GAPDH; **(C)**, the expression of Bcl-2/GAPDH. The loading control was GAPDH. All data shown are representative of three independent experiments with similar results. The statistical significance was analyzed using one-way ANOVA. CTX, cyclophosphamide-induced immunosuppressive group; LHT, levamisole hydrochloride group; AGRL, American ginseng red low dose group; AGRH, American ginseng red high dose group; AGSL, American ginseng soft branch low dose group; AGSH, American ginseng soft branch high dose group. ^##^ *P* < 0.01 vs. Control group, ^###^ *P* < 0.001 vs. Control group, ^***^ *P* < 0.01 vs. CTX group, ^&^ *P* < 0.05 vs. AGS group, ^&&^ *P* < 0.01 vs. AGS group, ^&&&^ *P* < 0.001 vs. AGS group, NS: no significant difference.

According to **Figure 5**, the expression of BAX was significantly increased, while that of Bcl-2 was significantly decreased in the CTX group compared to the control group, indicating that CTX caused apoptosis in the spleens of mice. Compared with the CTX group, the expression of BAX was significantly decreased, while that of Bcl-2 was significantly increased in the spleens of mice in the AGR and AGS groups. Compared with AGS, AGR significantly inhibited the protein expression of BAX and promoted the expression of Bcl-2 in the spleens of mice. Steamed American ginseng significantly inhibited apoptosis of splenic cells in mice, and the effect was better after processing.

### 3.6 AGR and AGS ameliorate CTX-induced immune deficiency by regulating the MAPK pathway

**Figure 6:**
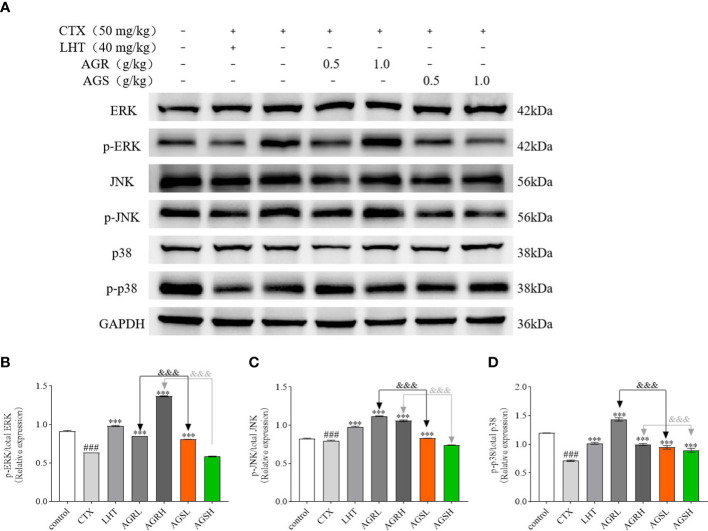
The protein expression of the MAPK signaling pathway in the spleen (N = 8, means ± SEM). **(A)**, images of the bands from the Western blotting; **(B)**, the relative expression of p-ERK/ERK; **(C)**, the relative expression of p-JNK/JNK; **(D)**, the relative expression of p-P38/P38. The load control was GAPDH. All data shown are representative of three independent experiments with similar results. The statistical significance was analyzed using one-way ANOVA. CTX, cyclophosphamide-induced immunosuppressive group; LHT, levamisole hydrochloride group; AGRL, American ginseng red low dose group; AGRH, American ginseng red high dose group; AGSL, American ginseng soft branch low dose group; AGSH, American ginseng soft branch high dose group. ^###^ *P* < 0.001 vs. Control group, ^***^ *P* < 0.001 vs. CTX group, ^&&&^ *P* < 0.001 vs. AGS group.

To assess the molecular mechanism by which American ginseng aqueous extracts mediated the regulation of immune-related proteins, we determined the expression of the MAPK signaling pathway. The MAPK family includes three major subgroups: p-ERK, p-JNK, and p-P38. Compared to the control group, the expression of p-JNK, p-ERK, and p-P38 was significantly lower in the spleens of mice in the CTX group in **Figure 6**. Compared with the CTX group, the expression of p-JNK, p-ERK, and p-P38 was significantly higher in the spleens of mice in the AGR and AGS groups. Compared with the AGS group, the expression of p-JNK, p-ERK, and p-P38 was significantly higher in the spleens of mice in the AGR group. AGR and AGSL could improve the immune activity of mice by activating the intracellular MAPK signaling pathway, and the effect was better after steaming.

## 4 Discussion

Enhancing human immunity to prevent and treat COVID-19 has emerged as a new topic in medicine, and herbal medicines play a crucial part in COVID-19 prevention and treatment. The outbreak of COVID-19 poses a severe threat to human health. As natural immunomodulators, herbs enhance the immune response to pathogens by activating immunoreactive cells (33). Under immune deficiency or overactivity, herbs can also enhance or suppress the immune response and restore the immune system to a healthy state (34). Natural immune reactions of plant origin are more stable with fewer side effects than chemically synthesized immunomodulators (35, 36), and more safe and effective natural immunomodulators should be developed to enhance the immunity against harmful chronic diseases, such as stress, obesity, ischemia, tuberculosis, diabetes, autoimmune diseases, and COVID-19 (37). American ginseng has received much attention in natural immunomodulator development because of its unique biological properties, such as antihypertensive (38), antioxidant (39), antitumor (40), antidiabetic, and cardiovascular disease prevention (41). Numerous American ginseng extracts have shown notable immune-enhancing properties (11, 42–44). To demonstrate the immunomodulatory activity of American ginseng, Yu et al. (45) extracted alkali-extractable polysaccharide from an aqueous extract of American ginseng, which significantly increased the expression of NO, TNF-α, and IL-6 in macrophages. Wang et al. (46) prepared North American ginseng extracts (CVT-E002), which increased the splenic B cells and the expression of serum immunoglobulin. The most popular animal model in the research concerning the stimulatory and regulatory effects of bioactive substances on the immune system is CTX-induced immunosuppression in male BALB/c mice (47, 48). In this study, LHT was used as a positive control (49, 50), and male BALB/c mice with CTX-induced immunosuppression served as an animal model to investigate the regulatory potential of AGR and AGS on CTX-induced immunosuppression in male BALB/c mice and evaluate whether steam-processing increased the immunomodulatory activity.

Immune tissues, immune organs, immunological cells, and immune active chemicals consist of the immune system. In some cases, the spleen and thymus are crucial immunological organs controlling the immune responses in the body, and the spleen and thymus indices can directly reflect non-specific immune activity (51). First, LHT, AGR, and AGS can significantly reverse the body weight loss and immune organ index declines induced by CTX. Additionally, the splenic vesicles were improved, suggesting that LHT and American ginseng can repair the spleen tissue damage induced by CTX. Furthermore, body weight and immune organ indices were significantly increased. Lymphocytes are considered a crucial defense against invasive infections (52), and T lymphocyte proliferation is a crucial cellular immunity index (53). B cells produce antibodies and increase the capacity of cellular immune response with the assistance of T helper cells (Th). Activated Th cells generate cytokines that control the activity of T cells, B cells, monocytes, macrophages, and other immune cells. Th cells can be divided into three groups based on cytokine differences: Th_1_, Th_2_, and Th_0_. Th_0_ lymphocytes primarily produce IL-2 but can secrete cytokines with Th_1_ and Th_2_ properties. In addition to secreting IFN-γ, IL-2, and TNF-α, Th_1_ activates T cells and monocytes, promotes T cell-mediated cellular immunity, and stimulates B cell IgM and IgG synthesis (54, 55). Th_2_ secretes IL-2 and TNF-α, triggers IgG and IgM conversion on B cells, and controls humoral immunity (56). It works to trigger allergic immune reactions when IFN-γ is relatively absent.

Cytokines and immunoglobulins are vital to immunity (57, 58). Cytokines are low molecular weight soluble proteins with extensive biological activities secreted by immune cells and some non-immune cells, such as chemokines, oncogenes, and growth factors (59). These play various roles in immunity, including intrinsic and adaptive immunity regulation, cell growth and proliferation, and damaged tissue repair. Produced by various cells, including immune cells, endothelial cells, and mast cells, TNF-α directly kills or induces apoptosis of target cells. During active innate immunity, immune cells rapidly produce TNF (60). IFN-γ is primarily produced by NK and Th_1_ cells, which stimulates macrophage phagocytic activity, mediates cellular immune function, promotes Th_1_ cell differentiation and proliferation, encourages IgG production, and activates complement (61), constituting a component of innate and antigen-specific immunities (62, 63). IL-2 plays a central role in the maturation and development of lymphocytes and monocytes as a T-cell growth factor (64). IgA is the most important antibody for mucosal immunity, IgG is the most common antibody subtype found in serum, and IgM is the largest antibody subtype in terms of molecular weight (65). CTX negatively affects monocytes and macrophages, decreases the proliferation rate of T lymphocytes, and reduces the ratio of CD4^+^CD8^-^/CD4^-^CD8^+^. CTX also inhibits the serum levels of TNF-α, IL-2, IFN-γ, IgA, IgM, and IgG in mice (66–68). The number of CD4^+^CD8^-^T cells and the proliferation rate of T lymphocytes in the mice spleen were significantly increased in the AGRH group, whereas the AGRL and AGS groups only showed significantly increased proliferation rate of T lymphocytes, without significantly regulated T lymphocyte subsets. LHT, AGR, and AGS groups showed significantly increased expression of IL-2, IFN-γ, TNF-α, IgA, IgG, and IgM. Moreover, AGR greatly improved the expression of IgA, TNF-α, and IFN-γ compared to AGS. Thus, AGR had improved immunomodulatory effects on mice with CTX-induced immunosuppression by significantly increasing the expression of cytokines and immunoglobulins.

The Bcl-2 family is essential in controlling the mitochondrial apoptotic pathway (69). Bcl-2 is a key anti-apoptotic protein (70), and BAX is a key pro-apoptotic protein (71). MAPK is a class of serine/threonine protein kinases highly conserved in eukaryotic species, serving as central signaling elements regulating cell proliferation, differentiation, and stress responses (72–74). They are also known as classical pathways that regulate immune responses (75). MAPK is linked to T cell development and function, such as ERK required for T cell proliferation and CD4 T cell polarization. ERK and JNK are also important upstream regulators of IL-2 transcription, and the decrease in the expression of IL-2 production may be due to the decreased expression of ERK and JNK (76–80). Additionally, the activation of ERK, JNK, and P38 is necessary for CD8T cells to respond in a cytotoxic manner (81). P38MAPK can increase the stability and translation of cytokine mRNA, which can increase cytokine levels. P38 is also a crucial regulator of the IFN produced by CD4 and CD8 T cells (82, 83). CTX promotes the expression of Bax, suppresses the expression of Bcl-2 (84, 85), and inhibits the phosphorylation of the MAPK pathway (86). In this study, LHT, AGR, and AGS significantly reduced the expression of BAX, increased the expression of Bcl-2, and activated the expression of p-JNK, p-ERK, and p-P38 proteins compared to the CTX group. The findings demonstrated that steaming significantly increased the immunomodulatory effect of American ginseng in reducing CTX-induced immunosuppression in mice.

Red ginseng is a popular processed form, whereas steamed American ginseng is increasingly common. Homologous herbs of American ginseng, including Asian ginseng, have likewise been demonstrated to actively and passively improve immunity (30, 87). Compared to white ginseng, ginseng steamed at high temperatures has a different chemical composition and much more free radical scavenging action (88). Heat-processed ginseng significantly increased cytokine expression and activated MAPK and NF-κB pathways in RAW264.7 cells compared to white ginseng (89). Saba et al. investigated the antioxidant and immunostimulatory activities of red ginseng, black ginseng, and fermented red ginseng using an acetaminophen-induced oxidative stress model and a cyclophosphamide-induced immunosuppression model, concluding that red ginseng had strong antioxidant and immunostimulatory activities compared to black ginseng and fermented red ginseng (90). The pharmacological action of herbs is affected by the processing methods. Steaming of botanicals provides new techniques to boost immunity and cure immune-related disorders.

## 5 Conclusion

This study investigated the effects of steaming on American ginseng’s immunomodulatory properties. The results showed that AGR and AGS could relieve CTX-induced immunosuppression by enhancing immune organ indices, improving cell-mediated immune response, increasing serum cytokine and immunoglobulin levels, and promoting macrophage activities such as carbon clearance and phagocytic index. Compared to AGS, AGR significantly improved the number of CD4 T cells, the spleen indices, and the levels of IgA, IgG, TNF-α, and IFN-γ in serum, markedly increasing the expression of the ERK/MAPK pathway. AGR may be an effective immunomodulatory agent capable of preventing immune system hypofunction. Future research may investigate the exact mechanism to rule out any unforeseen effects of AGR and AGS.

## Data availability statement

The original contributions presented in the study are included in the article/supplementary files, further inquiries can be directed to the corresponding authors.

## Ethics statement

The animal study was reviewed and approved by the Institute of Research Special Animal and Plant Sciences, China Academy of Agricultural Sciences.

## Author contributions

Y-TZ and Y-SS conceived the study and designed the project. Y-TZ performed the animal experiment and manuscript. J-YS and X-HH performed the data analysis. D-DR and YZ collected the samples. WT, Y-SL, Z-ML, S-SL, and Y-SS revised the manuscript and supervised the whole study. All authors contributed to the article and approved the submitted version.
